# The effects of acute cannabidiol on cerebral blood flow and its relationship to memory: An arterial spin labelling magnetic resonance imaging study

**DOI:** 10.1177/0269881120936419

**Published:** 2020-08-07

**Authors:** Michael A P Bloomfield, Sebastian F Green, Chandni Hindocha, Yumeya Yamamori, Jocelyn Lok Ling Yim, Augustus P M Jones, Hannah R Walker, Pawel Tokarczuk, Ben Statton, Oliver D Howes, H Valerie Curran, Tom P Freeman

**Affiliations:** 1 Translational Psychiatry Research Group, Research Department of Mental Health Neuroscience, Division of Psychiatry, Institute of Mental Health, University College London, London, UK; 2 Clinical Psychopharmacology Unit, Research Department of Clinical, Educational and Health Psychology, University College London, London, UK; 3 Psychiatric Imaging Group, Medical Research Council London Institute of Medical Sciences, Imperial College London, Hammersmith Hospital, London, UK; 4 NIHR University College Hospitals London Biomedical Research Centre, University College London, London, UK; 5 The Traumatic Stress Clinic, St Pancras Hospital, Camden and Islington NHS Foundation Trust, London, UK; 6 National Hospital for Neurology and Neurosurgery, University College London Hospitals NHS Foundation Trust, London, UK; 7 Medical Research Council London Institute of Medical Sciences, Imperial College London, Hammersmith Hospital, London, UK; 8 Psychosis Studies Department, Institute of Psychiatry, Psychology and Neuroscience, King’s College London, London, UK; 9 Department of Psychology, University of Bath, Bath, UK; 10 Clinical Psychopharmacology Unit, Research Department of Clinical, Educational and Health Psychology, University College London, London, UK

**Keywords:** ASL, cannabidiol, hippocampus, memory, MRI, perfusion

## Abstract

**Background:**

Cannabidiol (CBD) is being investigated as a potential treatment for several medical indications, many of which are characterised by altered memory processing. However, the mechanisms underlying these effects are unclear.

**Aims:**

Our primary aim was to investigate how CBD influences cerebral blood flow (CBF) in regions involved in memory processing. Our secondary aim was to determine if the effects of CBD on CBF were associated with differences in working and episodic memory task performance.

**Methods:**

We used a randomised, crossover, double-blind design in which 15 healthy participants were administered 600 mg oral CBD or placebo on separate days. We measured regional CBF at rest using arterial spin labelling 3 h after drug ingestion. We assessed working memory with the digit span (forward, backward) and n-back (0-back, 1-back, 2-back) tasks, and we used a prose recall task (immediate and delayed) to assess episodic memory.

**Results:**

CBD increased CBF in the hippocampus (mean (95% confidence intervals) = 15.00 (5.78–24.21) mL/100 g/min, *t_14_* = 3.489, Cohen’s *d* = 0.75, *p* = 0.004). There were no differences in memory task performance, but there was a significant correlation whereby greater CBD-induced increases in orbitofrontal CBF were associated with reduced reaction time on the 2-back working memory task ( *r*= −0.73, *p* = 0.005).

**Conclusions:**

These findings suggest that CBD increases CBF to key regions involved in memory processing, particularly the hippocampus. These results identify potential mechanisms of CBD for a range of conditions associated with altered memory processing, including Alzheimer’s disease, schizophrenia, post-traumatic stress disorder and cannabis-use disorders.

## Introduction

Cannabidiol (CBD) is one of the main constituents of cannabis and is gaining interest for its broad therapeutic potential (Campos et al., 2016; Devinsky et al., 2014; Freeman et al., 2019; Zuardi, 2008). In addition to antipsychotic (Leweke et al., 2012; McGuire et al., 2018; Zuardi et al., 2012) and anxiolytic properties (Bergamaschi et al., 2011; Blessing et al., 2015; Crippa et al., 2011; Soares and Campos, 2017), there is some evidence to suggest that CBD may improve memory impairment across multiple domains, including working and episodic memory, as demonstrated in several preclinical models (Avraham et al., 2011; Barichello et al., 2012; Campos et al., 2015; Cassol et al., 2010; Cheng et al., 2014a, 2014b; Fagherazzi et al., 2012; Magen et al., 2009, 2010; Martin-Moreno et al., 2011; Pazos et al., 2012; Schiavon et al., 2014; Wright et al., 2013), cannabis users (Morgan et al., 2010, 2012), and in cognitive impairment caused by the other main constituent of cannabis, ∆^9^-tetrahydrocannabinol (THC) (Englund et al., 2013; Hindocha et al., 2015), although this has not been found in all studies (Boggs et al., 2018; Hindocha et al., 2018a; Morgan et al., 2018). Additionally, CBD modulates emotional memory processing (Bitencourt and Takahashi, 2018; Das et al., 2013; de Carvalho and Takahashi, 2017; Hindocha et al., 2015; Hudson et al., 2018; Lee et al., 2017; Stern et al., 2017; Uhernik et al., 2018), which may help to explain its putative therapeutic effects in post-traumatic stress disorder (PTSD; Hindocha et al., 2019; Shannon and Opila-Lehman, 2016) and anxiety disorders. However, the precise mechanisms underlying the effects of CBD on memory are unclear.

There is evidence that CBD alters cerebral blood flow (CBF) (Crippa et al., 2004, 2011) and this offers one possible mechanism through which it may influence memory function. CBD has been widely described as an arterial vasodilator (Sultan et al., 2017), and increases CBF in mouse models of stroke (England et al., 2015). In human single-photon emission computed tomography (SPECT) studies of resting state, 400 mg of oral CBD modulated resting CBF in key limbic and paralimbic regions involved in memory processing, including decreased CBF in the left amygdala-hippocampal complex and increased CBF in the left parahippocampal gyrus in healthy volunteers (Crippa et al., 2004). A similar study from the same laboratory later found that CBD decreased resting CBF in the left hippocampus and parahippocampal gyrus in patients with anxiety disorder (Crippa et al., 2011). Several functional neuroimaging studies using blood oxygen level dependent (BOLD) functional magnetic resonance imaging (fMRI) have also demonstrated haemodynamic effects of CBD (Bhattacharyya et al., 2012a), including reductions in medial temporal lobe structures whilst viewing fearful faces (Fusar-Poli et al., 2009) and during attentional salience processing (Bhattacharyya et al., 2012b).

However, the proposed effects of CBD on regional CBF in humans have been disputed (Sultan et al., 2017). Previous studies (Crippa et al., 2004, 2011) have directly measured CBD-related changes in regional CBF using SPECT, an imaging modality with relatively low resolution to investigate regional effects. Additionally, no study has investigated the association between regional CBF and memory task performance under acute CBD. Our primary aim was therefore to investigate the acute effects of CBD on CBF in regions involved in memory processing in healthy individuals at rest using arterial spin labelling (ASL), a non-invasive, direct measure of CBF. Our secondary aim was to investigate the relationship between CBF and memory performance in episodic and working memory tasks. We defined regions of interest (ROIs) in the medial temporal lobe (MTL) and prefrontal cortex (PFC) a priori, which are differentially involved in both memory domains, including the hippocampus (Leszczynski, 2011; Squire and Zolamorgan, 1991), parahippocampal gyrus (Luck et al., 2010; Zolamorgan et al., 1989), amygdala (Hamann et al., 1999; Peinadomanzano, 1990; Phelps, 2004), dorsolateral PFC (Mars and Grol, 2007; Nyberg et al., 1996), orbitofrontal cortex (OFC) (Barbey et al., 2011; Brand and Markowitsch, 2006) and ventromedial PFC (Bechara et al., 1998; Bonnici et al., 2012). Based on previous studies, we hypothesised that CBD would decrease resting CBF in the hippocampus. We also sought to explore the differences in CBF in each of the other ROIs described above. Finally, we explored the relationship between regional CBF and memory performance.

## Materials and methods

This study was conducted in accordance with Good Clinical Practice and the Helsinki Declaration (UCL Research Ethics Committee 3325/002). Participants provided written informed consent and received an honorarium for participation (£10 per hour).

### Study participants

Participants were recruited through online adverts, posters and word-of-mouth. All participants included were right-handed and aged 18–70 (see Table 1 for demographic and clinical characteristics). Exclusion criteria were: (a) current use of psychotropic agents; (b) current or past use of cannabis or CBD; (c) previous use of any psychoactive (recreational) drug on >5 occasions; (d) current or previous mood disorder, psychosis, anxiety disorder, or substance abuse disorder according to Diagnostic and Statistical Manual of Mental Disorders IV (DSM-IV) criteria; (e) current nicotine dependence (defined by Fagerström Test for Nicotine Dependence (Heatherton et al., 1991)); (f) score >7 on the Alcohol Use Disorders Identification Test (Saunders et al., 1993); (g) pregnancy; (h) impaired mental capacity; (i) allergy to CBD or placebo excipients; (j) claustrophobia or other contraindications to MRI.

**Table 1.** Demographic and baseline clinical characteristics of study participants (*n* = 15).

Characteristic	*n*
Sex	nine female, six male
	**Mean (±SD)**
Age (years)	24.1 (±5.0)
BMI (kg/m^2^)	22.6 (±4.1)
AUDIT score (0–40)	1.5 (±1.7)
FDNT score (0–10)	0.0 (±0.0)
BAI score (0–63)	2.5 (±3.9)
BDI score (0–63)	1.4 (±2.0)
ASI score (0–29)	2.1 (±3.1)

ASI: addiction severity index (averaged across four readings over the two sessions); AUDIT: alcohol use disorders identification test; BAI: beck anxiety inventory; BDI: beck depression inventory; BMI: body mass index; FDNT: *Fagerström* test for nicotine dependence.

### Study design

We used a within-subjects, randomised, double-blind, placebo-controlled design. Participants received single doses of either 600 mg of CBD (pure synthetic (-)-CBD) or placebo in identical capsules at two sessions, separated by at least one week. Synthetic CBD (99.9% purity) was obtained from STI Pharmaceuticals (Brentwood, UK) and manufactured by Nova Laboratories (Leicester, UK). Size 2 gelatin capsules contained microcrystalline cellulose filler and CBD. Matched placebo capsules contained lactose filler. Whilst earlier SPECT studies used a lower dose of 400 mg (Crippa et al., 2004, 2011), more recent fMRI studies demonstrate that 600 mg is safe and exhibits measurable perfusion changes (Bhattacharyya et al., 2012a); a larger dose was opted on the assumption that it would be associated with larger, more measurable effects. The order of drug was randomised and stratified for sex. Following drug administration, brain scanning occurred at +180 min post capsule ingestion, which coincides with previously described peak plasma concentrations (Haney et al., 2016), and memory tasks were performed in succession roughly from between +275 and +340 min (see Supplemental material). Participants underwent three memory tasks: a prose recall task, N-back task and digit span task, and completed other tasks to be reported elsewhere. Matched versions of the tasks were used in the two study sessions. In order to control for variation in the absorption of CBD, participants were instructed to fast from midnight (excluding water, and caffeine if part of their morning routine) until after the brain scanning session. All participants underwent neuroimaging at least 12 h since their last meal; time of last meal was confirmed as part of pre-test screening conducted on the morning of testing. Drug administration and neuroimaging occurred at the same time each session. Drug administration was at 09:00 and neuroimaging at 12:00.

### Power calculation

Two previous human studies have found acute effects of CBD v. placebo on resting CBF using a sample size of *n* = 10 in a crossover design (Crippa et al., 2004, 2011). This study has a sample size of *n* = 15, providing a 50% increase in sample size from these previous studies to adjust for Winner’s Curse (Button et al., 2013; Hindocha et al., 2018b). A sensitivity power analysis conducted using G*Power 3 (Faul et al., 2007) indicated that our sample size would provide 80% power to detect a large effect size (Cohen’s *d* = 0.8) at an alpha of 0.05.

### Memory assessments

#### Prose recall task

The prose recall subtest of the Rivermead Behavioural Memory test (Wilson et al., 1991) taps episodic memory. Participants heard 30 s of prose (a news bulletin) and recalled it immediately and following a 25 min delay filled with other assessments, which included other tasks to compete as a distractor. The number of idea units recalled out of 21 was recorded.

#### N-back task

The N-back task (Freeman et al., 2012; Hindocha et al., 2017; van der Wee et al., 2003[TS: please link van der Wee to reference]), a spatial working memory (WM) task, required participants to observe sets of visual stimuli in one of six locations in a sequential order, and then record when the current stimulus corresponds with the stimulus seen in a pre-defined region (0-back), one step earlier (1-back), and two steps earlier (2-back). This order was fixed, and participants had a practice session before each N-back stage. Reaction time (RT) and accuracy were recorded automatically.

#### Digit span task

In the digit span task (Wechsler, 1997), which taps WM, participants read a series of digit strings to participants, who were then required to recall the digits in the same order in which they appeared, both forwards and backwards. Forwards recall taps maintenance of digits, where backwards recall taps both maintenance and manipulation of digits (Aben et al., 2012). The number of items increased every two strings starting from three for forwards and two for backwards. If a participant failed both strings at each level (i.e. strings of four numbers), the task was terminated, and moved on to backward or ended. The number of digits correctly recalled was recorded. There were a maximum of 12 forwards and 12 backwards series.

### Image acquisition

We conducted image acquisition and data analysis blind to drug condition. We used a Siemens Magnetom Prisma 3T scanner to perform 3D axial pulsed ASL using the FAIR-QUIPSS II (Flow-sensitive Alternating Inversion Recovery–QUantitative Imaging of Perfusion Using a Single Subtraction) acquisition method (Wong et al., 1998). Scanner parameters were as follows: background suppression (grey-white) was on, bolus length (BL) 700 ms, inversion time (TI) 1990 ms, TR 4600 ms, TE 13.36 ms, slice thickness 4 mm, flip angle 180°, voxel size (mm) 1.9 × 1.9 × 4.0, bandwidth 3256 Hz/pixel, presumed tissue blood partition coefficient of water (λ) 0.9 mL/g, presumed relaxation time of blood (*T*_1b_) 1650 ms, inversion fraction (α) 0.98. Movement-corrected perfusion maps were calculated from the control and label images, and separately acquired M_o_ maps, using a MATLAB (Mathworks, Inc.) script written in-house (see Supplemental material).

Structural images were acquired using a T1 MPRAGE with 1 mm^3^ isotropic voxels, TR = 2300 ms, TE = 2.91, TI = 900, flip angle = 9°, parallel imaging factor = 2, bandwidth = 140 Hz/pixel. Using fMRIB Linear Image Registration Tool (FLIRT) within fMRIB Software Library (FSL) perfusion maps were registered to structural images and converted into standard Montreal Neurological Institute (MNI) space. Masks were generated using the Harvard–Oxford probabilistic atlas and applied to the specified regions of interest (ROI). Mean values of CBF (mL/100 mL/min) were then extracted using FSL.

### Plasma CBD concentrations

We performed venepuncture immediately after scanning to measure CBD concentrations. Blood samples were collected in EDTA vacutainers and were immediately centrifuged to plasma for storage at −80°C. Samples were analysed using Gas Chromatography coupled with Mass Spectrometry with a lower limit of quantification of 0.5 ng/mL.

### Statistical analysis

Two-tailed paired *t*-tests (significance threshold *p* <0.05, Holm–Bonferroni corrected for family-wise errors across six bilateral regions-of-interest) were performed to compare CBF between CBD and placebo sessions (i.e. both drug conditions) for each region. Repeated measures ANOVAs with a factor of drug (CBD v. placebo) were used to compare performance on memory tasks. For the N-back, an additional factor of load (0-back, 1-back, 2-back) was included for the outcomes of accuracy and RT. For the digit span, the additional factor was direction (forward, backward). For the prose recall task, the additional factor was delay (immediate, delayed). All post-hoc pairwise comparisons were Bonferroni-corrected. To investigate the relationship between differences in regional CBF and memory task performance, correlations were performed using the Pearson correlation coefficient at a two-tailed significance threshold of *p* <0.005 adjusted for 10 simultaneous comparisons using Bonferroni correction. This was performed for regions which demonstrated statistically significant differences in CBF (*p* <0.05). The 10 simultaneous comparisons were correlations between differences in hippocampal or orbitofrontal perfusion and differences in N-back accuracy and reaction time at 0-back, 1-back and 2-back, differences in forward and backward digit span scores, and differences in delayed and immediate prose recall scores. To assess differences in plasma CBD concentrations between the CBD and placebo groups, the non-parametric Wilcoxon signed ranks test was used. Levels of plasma CBD were correlated against hippocampal and orbitofrontal CBF during the CBD session using the Pearson correlation coefficient. Each analysis was repeated with a between subject factor of order of drug which did not significantly change the results; therefore, results are displayed without this factor.

## Results

### Demographic and clinical characteristics

Participant demographics and baseline clinical characteristics are in Table 1. From an original cohort of 17 healthy participants, we excluded two due to excessive rotation or motion artefact (voxel shift >9 mm and rotation >2°) resulting in 15 participants (nine female, six male).

### Regional cerebral blood flow

**Figure 1.**
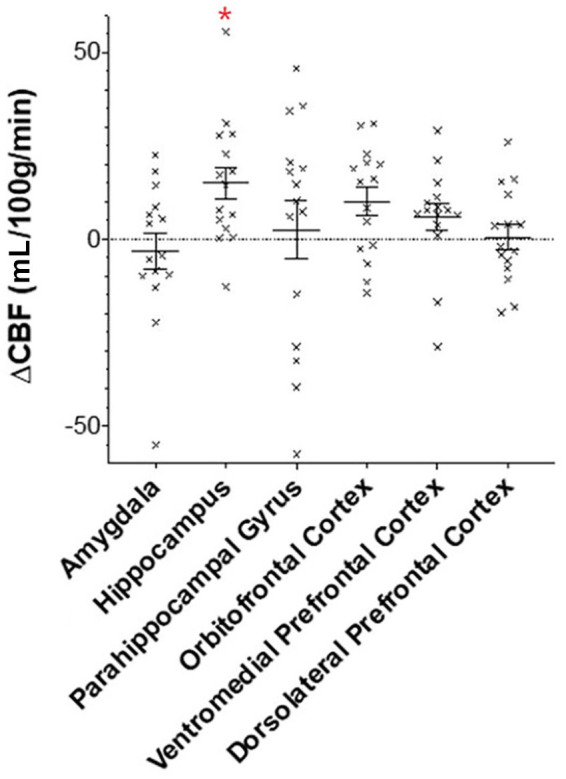
Differences in regional CBF between CBD and placebo. ΔCBF is the mean difference in cerebral blood flow (mL/100 g/min ± standard error of the mean) after CBD v. placebo in regions within the medial temporal lobe and prefrontal cortex. The asterisk indicates regions with statistically significant change in CBF after correcting for multiple comparisons.

**Figure 2.**
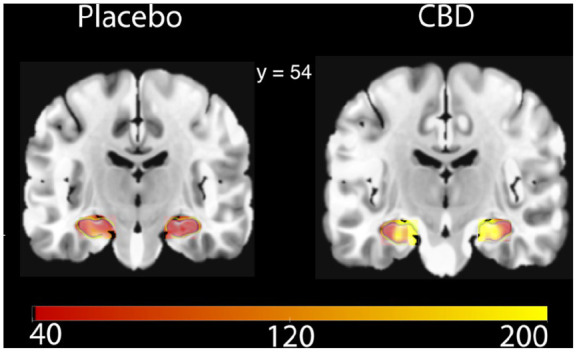
Differences in hippocampal CBF after CBD or placebo. This coronal slice highlights differences in grey matter perfusion in the hippocampus (in green). Scale bar units = mL/100 g/min. MNI coordinate displayed.

See Table 2 and Figure 1 for differences in regional CBF between CBD and placebo. CBD caused a significant increase in CBF to the hippocampus (mean difference 15.00 mL/100 g/min (95% confidence intervals (CI) 5.78–24.21, *t_14_* = 3.489, *p* = 0.004, Cohen’s *d* = 0.75, Figure 2) which survived Holm–Bonferroni correction. CBD did not cause significant differences in CBF in other regions of the MTL. In the PFC, CBD caused a significant increase in CBF in the OFC by a mean difference of 10.04 mL/100 g/min (95% CI 1.90–18.19, *t_14_* = 2.644, *p* = 0.019, Cohen’s *d* = 0.55), however, this did not survive Holm-Bonferroni correction. There were no significant differences in other PFC areas. There were no significant effects of controlling for drug order on CBF in all regions.

**Table 2.** Differences in regional cerebral blood flow between CBD and placebo.

Region of interest	Δ Perfusion (mL/100 g/min (CI))	Δ Perfusion (% (CI))	*t_14_* statistic	Cohen’s *d*	*p*	*p* ^#^
Hippocampus	15.00 (5.78–24.21)	12.69 (5.75–19.63)	3.489	0.75	0.004**	0.024*
Parahippocampal gyrus	2.52 (−14.33–19.38)	−0.32 (−13.87–13.23)	0.792	0.09	0.441	1.000
Amygdala	−3.26 (−13.80–7.29)	−4.31 (−16.48–7.86)	−0.663	0.15	0.518	1.000
Orbitofrontal cortex	10.04 (1.90–18.19)	7.02 (1.06–12.97)	2.644	0.55	0.019*	0.095
Ventromedial prefrontal cortex	5.95 (−1.69–13.60)	4.55 (−2.76–11.85)	1.671	0.20	0.117	0.468
Dorsolateral prefrontal cortex	0.60 (−6.51–7.70)	−0.73 (−7.55–6.08)	0.180	0.02	0.860	0.860

CI: confidence interval.

* Significant at the *p* <0.05 level.

** Significant at the *p* <0.01 level.

*p*^#^ = Holm–Bonferroni corrected (using alpha 0.05).

### Memory task performance

**Figure 3.**
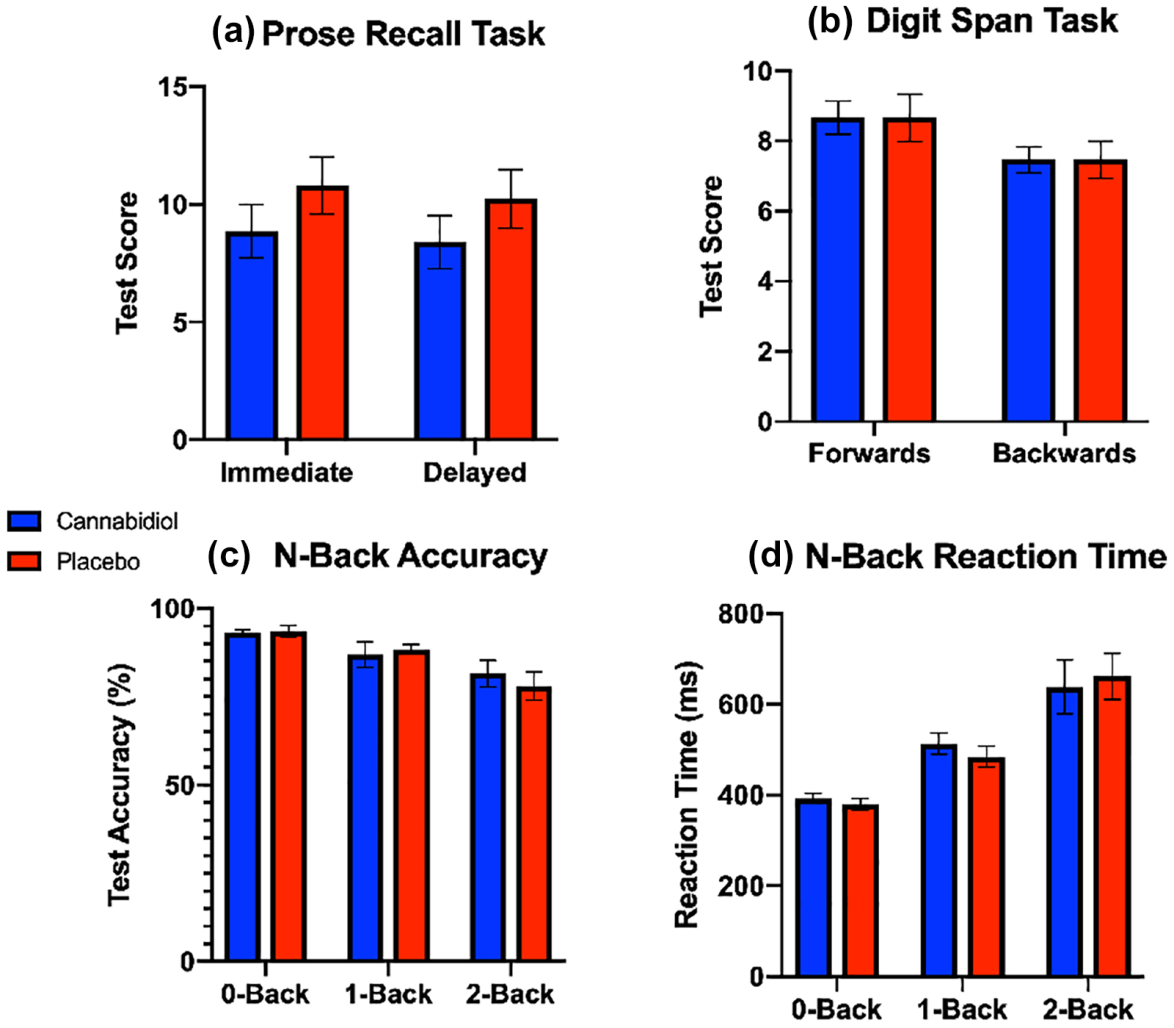
Differences in memory task performance in the (a) prose recall, (b) digit span, and (c, d) N-back task (mean ± standard error of the mean) post-CBD or placebo.

For the prose recall task, there was no main effect of drug (*F_1,14_* = 3.701, *η*^2^ = 0.184, *p* = 0.075, mean difference −0.517, 95% CI −1.126–0.092, Figure 3a), or task (immediate or delayed; *F_1,14_* = 3.311 *η*^2^ = 0.014, *p* = 0.090), and there was no significant drug*task interaction (*F_1,14_* = 0.037, *η*^2^ = 0.000, *p* = 0.850).

For the digit span task, there was no main effect of drug (*F_1,14_* = 0.312, *η*^2^ = 0.007, *p* = 0.585), there was a significant effect of task (forwards vs backwards; *F_1,14_* = 9.333, *η*^2^ = 0.182. *p* = 0.009) reflecting the standard decreased score in backwards digit span score compared with forwards (see Figure 3b), but there was no significant drug–task interaction (*F_1,14_* = 0.497, *η*^2^ = 0.007, *p*= 0.492).

Two participants did not complete all parts of the N-back task and were therefore removed from analysis. For accuracy scores in the N-back task, there was no main effect of drug (*F_1,12_* = 0.026, *η*^2^ = 0.000, *p* = 0.875), there was a significant effect of task (0-back *vs.* 1-back v. 2-back; *F_2,24_* = 10.180, *η*^2^ = 0.305, *p* = 0.001) shown by decreasing accuracy across increasing WM load (Figure 3c), but there was no drug*task interaction (*F_2,24_*=0.693, *η2* =0.016, *p*=0.510). For RTs in the N-back task, there was no main effect of drug (*F_1,12_*=0.168, *η*^2^=0.000, *p*=0.689), there was a main effect of task (0-back *vs.* 1-back *vs.* 2-back; *F_1,12_*=25.642, *η*^2^=0.619, *p*<0.001) as shown by increasing RTs across increasing WM load (Figure 3d) but there was no drug–task interaction (*F_2,24_* = 1.420, *η*^2^ = 0.006, *p* = 0.261). All analyses for memory task performance were repeated with a between subjects’ factor of order of drug administration and the results did not change.

Correlational analysis showed that CBD-induced differences in OFC CBF were correlated with 2-back task performance after correcting for multiple comparisons, such that increased CBF in the OFC following CBD was associated with decreased RT and thus better working memory performance (*r_11_* = −0.73, *p* = 0.005; Supplemental Figure 1). There were no other statistically significant relationships between OFC CBF and 0-back and 1-back conditions, digit span, or prose recall tasks. There were no statistically significant correlations between changes in hippocampal CBF and memory performance after correcting for multiple comparisons. There was no significant effect of correcting for order of drug administration.

### Plasma CBD levels

Analysis of plasma CBD levels using Wilcoxon Signed-Ranks Test showed that the CBD session ranks (median 6.010 ng/mL) were significantly higher than the placebo session ranks (median 0.000 ng/mL; *Z* = 3.124, *p*= 0.002; Supplemental Figure 2). There was a significant correlation between hippocampal CBF and CBD plasma levels during the CBD session (*r* = 0.623, *p* = 0.01), but this relationship was driven by an outlier. Once removed, this correlation was non-significant (*r* = 0.075, *p* = 0.798). Removal of this outlier still yielded a significant difference in hippocampal CBF under CBD v. placebo conditions (*t_df_* = 3.54*13, p*= 0.004). There was no significant correlation between orbitofrontal CBF and CBD plasma levels during the CBD session (*r* = 0.445, *p* = 0.09). We note that one participant had an elevated cannabidiol level (4.94 ng/mL) during their placebo session. Considering that sessions were conducted at least one week apart, and the elimination half-life of cannabidiol is approximately 18–32 h (Devinsky et al., 2014), it is likely that this participant consumed CBD from a third-party source before their placebo session which may have included consuming cannabis. This participant was excluded, and analysis was re-conducted with no significant changes: there remained a significant difference in hippocampal CBF (*t_df_* = 3.215_13_, *p* = 0.007), and a significant correlation between OFC CBF and 2-back reaction times (*r* = 0.760, *p* = 0.004).

## Discussion

To our knowledge, this is the first study to find that acute CBD increases CBF in the hippocampus. This supports the view that CBD has region-specific haemodynamic effects in the human brain, which has previously been disputed (Sultan et al., 2017).

### Relation to previous studies

Our finding of increased hippocampal CBF after CBD contrasts with two previous SPECT studies (Crippa et al., 2004, 2011). These found a decrease in CBF in healthy (Crippa et al., 2004) and anxiety disorder participants (Crippa et al., 2011). There are several possible explanations for this difference. Our study had higher statistical power than the previous two studies, with increased sample size of 50% to account for inflation of effect size (Button et al., 2013; Hindocha et al., 2018b). Moreover, our study used ASL which has better resolution to detect regional effects than SPECT. Our findings are consistent with a meta-analysis reporting that CBD increased CBF in mouse models of stroke (Sultan et al., 2017). Additionally, we administered a higher dose (600 mg CBD) than previous studies (400 mg CBD; (Crippa et al., 2004, 2011) which may have accounted for different findings due to the complex dose-effect profile of CBD (Zuardi et al., 2017).

### Implications for hippocampal disorders

If replicated, the finding that acute CBD increases CBF in the hippocampus may be relevant for hippocampal disorders, since higher resting hippocampal blood flow is associated with better memory performance (Heo et al., 2010; Nishimura et al., 1998; Suzuki et al., 2016), although this relationship is not entirely clear (Finkelmeyer et al., 2016; Rane et al., 2013). With its key role in learning and memory (Sweatt, 2004), the hippocampus is an important therapeutic target across multiple neuropsychiatric disorders including schizophrenia (Green et al., 2000), depression (Rock et al., 2014), PTSD (Scott et al., 2015) and Alzheimer’s disease. Regional CBF is strongly associated with brain volume change and has a complex bidirectional relationship (Appelman et al., 2008; Zonneveld et al., 2015). Since acute CBD may increase hippocampal CBF, further studies are required to investigate whether CBD can attenuate the hippocampal structural alterations, including atrophy, and hippocampal-dependent memory impairments associated with these disorders (Ott et al., 2019). This notion is supported by findings that CBD can rescue hippocampal atrophy (Beale et al., 2018) and improve episodic memory performance (Englund et al., 2013) in chronic cannabis users. This finding may be particularly relevant to Alzheimer’s disease, where there are defects in blood flow control (Girouard and Iadecola, 2006).

The precise mechanisms through which CBD may modulate memory processing are unclear. In addition to causing endothelial vasodilatation through modulation of the endocannabinoid system (Stanley et al., 2015), CBD has numerous neuronal targets (Bih et al., 2015; Elsaid and Le Foll, 2020), which may underlie its pro-cognitive, anxiolytic, and antipsychotic effects (Premoli et al., 2019). Regarding memory, preclinical data suggest that CBD promotes hippocampal neurogenesis and facilitates synaptic plasticity (Campos et al., 2017). In addition, human spectroscopy studies demonstrate that CBD modulates glutamate and GABA levels across several limbic and prefrontal regions (Pretzsch et al., 2019). Through its cerebrovascular and neuronal activity, CBD may therefore alter the dysfunctional prefrontal-hippocampal circuitry associated with impaired memory in several neuropsychiatric disorders (Jin and Maren, 2015).

Although we found no differences in memory performance following CBD, improvements in hippocampal-dependent memory tasks with CBD have been described elsewhere, including improved performance in prose recall during cannabis use (Morgan et al., 2010), and delayed verbal memory following THC administration (Englund et al., 2013). However, findings have also been mixed in this area. CBD has previously not demonstrated improvements in memory performance following THC-challenge (Morgan et al., 2018), nicotine abstinence (Hindocha et al., 2018b), and in schizophrenia (Boggs et al., 2018). As our study was in healthy participants, it is possible that ceiling effects account for this lack of CBD-induced differences in memory task performance, and the lack of relationship between hippocampal CBF and memory task performance. This variability may additionally be due to drug dose, route of administration, memory test timing, and methodological variation across studies. A number of the studies on CBD-related improved memory performance were conducted on models of cognitive impairment due to systemic insult (e.g. sepsis), Alzheimer’s, or current cannabis users which may not emerge in studies of healthy volunteers.

We found a moderately sized, negative correlation between the effects of CBD on OFC CBF and 2-back RT. Among other functions (Rolls, 2004), the OFC is involved in WM, particularly emotion and/or face processing working memory (LoPresti et al., 2008; Ross et al., 2013). The direction of this correlation and its specificity to the 2-back task suggests that the effects of CBD on CBF in the OFC might be related to an increase in performance in manipulation of working memory. However, given that the effects of CBD were weak for increasing OFC CBF and did not show an improvement in 2-back task overall, further evidence would be needed to support this hypothesis.

### Strengths and limitations

Major strengths of our study are the use of robust methodology including double-blinding, within-subject design, randomization of CBD and placebo order, and the inclusion of plasma CBD levels. ASL has good reproducibility within subjects (Jiang et al., 2010), and suffers from less inter-subject variability than BOLD signal in fMRI (Liu and Brown, 2007). Furthermore, ASL is a more direct neuroimaging modality for measuring regional CBF than other MRI methods, such as fMRI, which may detect reactive hyperaemia driven by astrocyte signaling (Attwell et al., 2010). ASL also offers better spatial resolution than other neuroimaging modalities used for similar studies such as SPECT.

Although our study had higher statistical power than previous studies (Crippa et al., 2004, 2011) it was not well powered to detect medium or small effect sizes. This study used a single dose of CBD in healthy volunteers, which may not translate to the effects of repeated CBD dosing and the use of CBD for psychiatric disorders or cognitive impairments. Finally, as the memory tasks were conducted outside of the scanner, we were unable to investigate the effects of CBD on fMRI measures of task performance, and correlations between these measures and CBF.

## Conclusion

We found evidence that acute CBD causes a significant increase in regional CBF to the hippocampus. These findings may have implications for the potential use of CBD across a range of disorders associated with hippocampal dysfunction including Alzheimer’s disease, PTSD and depression.

## Supplemental Material

JOP_CBD_ASL_Supplementary_Material – Supplemental material for The effects of acute cannabidiol on cerebral blood flow and its relationship to memory: An arterial spin labelling magnetic resonance imaging study Click here for additional data file. Supplemental material, JOP_CBD_ASL_Supplementary_Material for The effects of acute cannabidiol on cerebral blood flow and its relationship to memory: An arterial spin labelling magnetic resonance imaging study by Michael A P Bloomfield, Sebastian F Green, Chandni Hindocha, Yumeya Yamamori, Jocelyn Lok Ling Yim, Augustus P M Jones, Hannah R Walker, Pawel Tokarczuk, Ben Statton, Oliver D Howes, H Valerie Curran and Tom P Freeman in Journal of Psychopharmacology
