# Supplementary material for: The effects of acute cannabidiol on cerebral blood flow and its relationship to memory: An arterial spin labelling magnetic resonance imaging study
Source: J Psychopharmacol. 2020 Aug 7;34(9):981–9. doi: 10.1177/0269881120936419 (PMC7436497; doi:10.1177/0269881120936419)
Supplement: JOP_CBD_ASL_Supplementary_Material – Supplemental material for The effects of acute cannabidiol on cerebral blood flow and its relationship to memory: An arterial spin labelling magnetic resonance imaging study [file JOP_CBD_ASL_Supplementary_Material.pdf]

Supplementary material

Supplementary methods

To correct for head movement, all images were co-registered to the first M_o map, using a rigid deformation model, with a mono-modal (sum of squared differences) cost function for the later M_o maps. Images were then either co-registered with a mono-modal cost function or a multi-modal (Mattes Mutual Information) metric to the control and label images. Best results were obtained by using the modulus of the spatial gradient of the source images to determine the moving-to-fixed transformation, which was then applied to the source images; this had the effect of guiding the co-registration by emphasising landmarks, without reducing the images entirely to their edges. No brain extraction (skull-stripping) was used or needed. Images were interpolated to uniform voxels in the slice direction before the co-registration step, and down-sampled back to the original slice locations immediately afterwards. The final perfusion map was calculated using the single inversion-time formula:

$$f = \frac{\lambda}{2\alpha BL}\frac{\Delta M}{M_{0b}} exp\left(\frac{TI}{T_{1b}}\right)$$

f	Perfusion
λ	Tissue-blood partition coefficient of water
TI	Inversion time
ΔM	The difference signal between control and label images
M_{0b}	Equilibrium magnetization (containing blood spins only)
T_{1b}	The T_1 of blood
α	Inversion fraction
BL	Bolus length

Supplementary Table 1. Timeline of events, including image acquisition and memory tasks.

**Time**	**Event**
+ 0 mins	Administration of CBD or placebo
+ 180 mins	Subjects undergo MRI scanning
+ 260 mins	Plasma CBD levels taken
+ 275 mins	Prose Recall (Immediate)
+ 280 mins	N-Back Task
+ 300 mins	Prose Recall (Delayed)
+ 320 mins	Digit Span Task

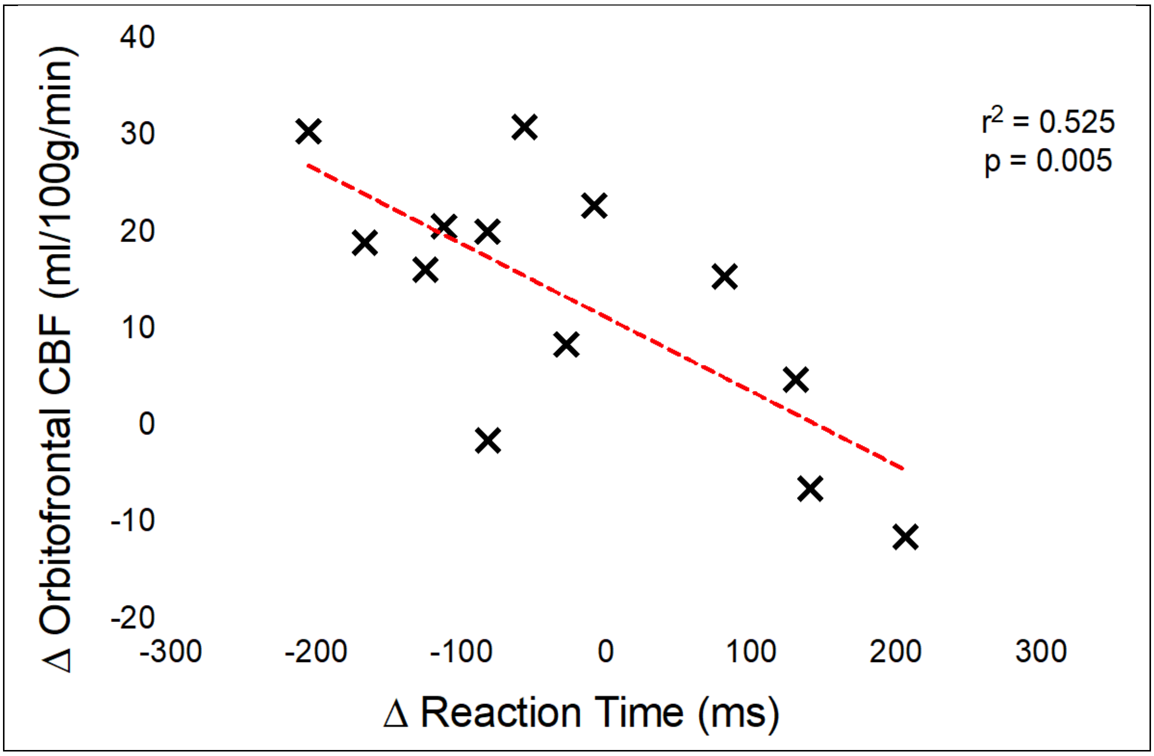

Supplementary Figure 1. The relationship between *differences* in blood flow to the orbitofrontal cortex and *differences* in reaction time in the 2-Back task post CBD vs. placebo

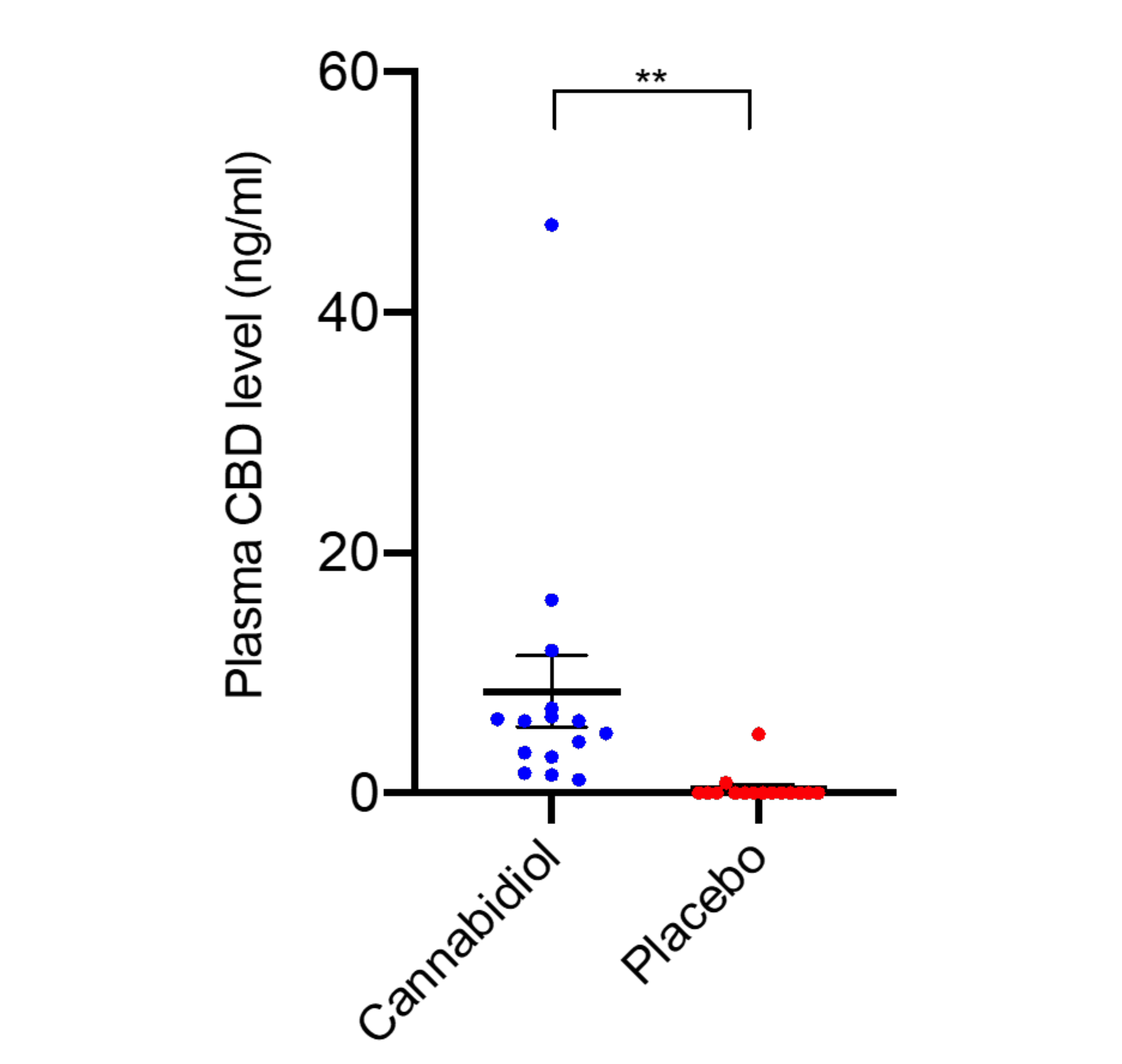

Supplementary Figure 2. Plasma CBD levels post-ingestion of 600 mg in CBD vs. placebo sessions. All data points are presented with the mean (±standard error of the mean). As demonstrated in this figure, the bioavailability of oral CBD is highly variable between individuals. (** = significant at the $p<0.01$ level
